# Adrenomedullin Improves Cardiac Remodeling and Function in Obese Rats with Hypertension

**DOI:** 10.3390/ph15060719

**Published:** 2022-06-06

**Authors:** Pei Qian, Qian Wang, Fang-Zheng Wang, Hang-Bing Dai, Hong-Yu Wang, Qing Gao, Hong Zhou, Ye-Bo Zhou

**Affiliations:** Department of Physiology, Nanjing Medical University, Nanjing 211166, China; peiqian@njmu.edu.cn (P.Q.); 2021110012@stu.njmu.edu.cn (Q.W.); fzwang@njmu.edu.cn (F.-Z.W.); hangbingdai@njmu.edu.cn (H.-B.D.); why971214@njmu.edu.cn (H.-Y.W.); gaoqing@njmu.edu.cn (Q.G.); hongzhou@njmu.edu.cn (H.Z.)

**Keywords:** adrenomedullin, obesity-related hypertension, heart, inflammation, oxidative stress, cardiac remodeling, cardiac function

## Abstract

This study aimed to determine whether adrenomedullin (ADM, 7.2 μg/kg/day, ip), an important endogenous active peptide, has a protective role in cardiac remodeling and function in obesity-related hypertension (OH) rats. A high-fat diet (HFD) was used to induce OH for 20 weeks. H9c2 cells incubated with palmitate (PA, 200 μM) to mimic high free fatty acid in obesity were used as an in vitro model. In OH rats, ADM not only decreased body weight (BW) and blood pressure (BP) but also improved systemic inflammation and oxidative stress. Moreover, ADM still had a greater inhibitory effect on local inflammation and oxidative stress in the hearts of OH rats, and the same anti-inflammatory and antioxidant effects were also confirmed in PA-treated H9c2 cells. The ADM receptor antagonist or Akt inhibitor effectively attenuated the inhibitory effects of ADM on inflammation and oxidative stress in PA-stimulated H9c2 cells. Furthermore, ADM application effectively normalized heart function, and hematoxylin-eosin and Masson staining and collagen volume fraction results showed that ADM improved cardiac remodeling in hearts of OH rats. ADM attenuated cardiac inflammation and oxidative stress via the receptor-Akt pathway, which involves the improvement of cardiac remodeling and function in OH rats.

## 1. Introduction

Visceral obesity is an important pathogenic factor in the pathogenesis of insulin resistance, leptin resistance, dyslipidemia, and hypertension, which increases the hazard of cardiovascular diseases [1,2]. Obesity-related hypertension (OH) is closely related to cardiovascular remodeling and dysfunction and contributes to the pathogenesis of chronic heart failure [2]. OH is characterized by multiple metabolic disorders, low-grade inflammation, oxidative stress, and hypertension, which promote adverse cardiac remodeling and injury, such as diastolic dysfunction [2,3,4]. Obesity not only raises blood pressure (BP) but also increases the left ventricular volume load and exacerbates inflammation and oxidative stress [5,6]. Circulating or local pro-inflammatory cytokines tumor necrosis factor-α (TNF-α), interleukin-1β (IL-1β) and IL-6, and reactive oxygen species (ROS) contribute to pathological hypertrophy by activating inflammatory signaling mitogen-activated protein kinase and nuclear factor kappa-B pathways and inhibiting the protein kinase B (Akt)/mammalian target of rapamycin (Akt-mTOR) pathway in cardiomyocytes; moreover, injured myocytes also promote localized cardiac inflammation and oxidative stress [6,7]. Therefore, the reduction of BP, and the local inhibition of cardiac inflammation and oxidative stress may be a good strategy to improve cardiac injury in OH. Therefore, new therapies for the treatment of OH-related cardiac abnormality that reduce morbidity and mortality are needed [8].

Endogenous adrenomedullin (ADM), a 52 amino acid peptide, plays important roles in both the health state and diseases, especially in the cardiovascular system [9,10,11]. The biological activity of ADM is mainly mediated by its receptor system through the combination of the receptor activity-modifying protein 2/3 (RAMP2/3) and calcitonin receptor-like receptor (CRLR). ADM receptors are widespread in the body, especially in cardiovascular tissues [12,13,14]. Previous studies have shown that ADM can decrease BP and increase cardiac output in normal rats [9]. Exogenous administration of ADM can protect against cardiac hypertrophy, cause a reduction in myocardial infarct size, and improve left ventricular remodeling and survival in some animal models [15,16,17,18,19,20]. More importantly, it exerts its protective roles in some animal models by inhibiting insulin resistance and anti-inflammatory and antioxidant mechanisms [21,22]. For instance, ADM reduces oxidative stress in injured cerebral tissue of rats [22] and inhibits inflammation of vascular endothelial cells of rats [23]. People with obesity display an obvious increase in ADM levels in plasma [24,25,26,27,28]. These findings suggest that ADM may have a critical role in OH rats by decreasing BP, and alleviating inflammation and oxidative stress in cardiac tissue to improve OH-related cardiomyopathy.

In the present study, we aimed to investigate the roles of ADM in the cardiac remodeling and function of rats with OH and related mechanisms. Therefore, this research was designed to explore whether ADM involved the hypertension and cardiac inflammation and oxidative stress of OH rats induced by a high-fat diet (HFD). Furthermore, the possible mechanisms of anti-inflammatory and antioxidant effects of ADM were investigated using an H9c2 cell line.

## 2. Results

### 2.1. The Roles of ADM in Body Weight, Hypertension, and Cardiac Parameters in Rats Fed by HFD

**Figure 1:**
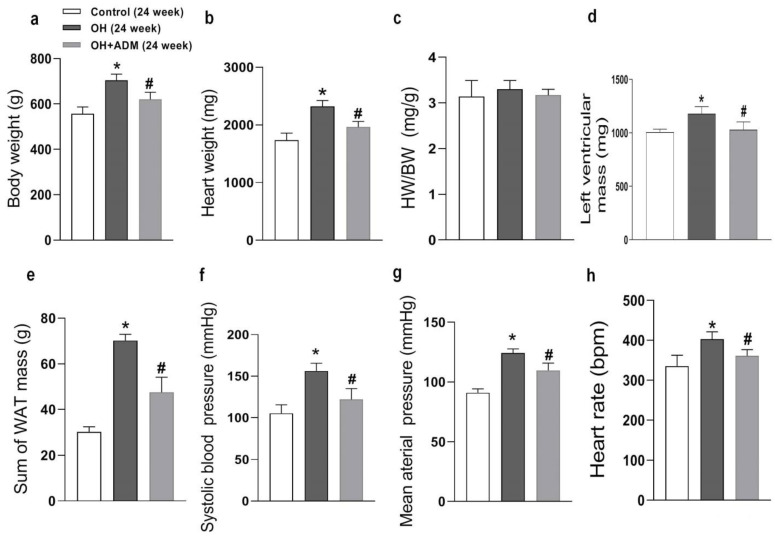
The anatomic data, systolic blood pressure (SBP), mean arterial pressure (**g**), and heart rate after 24 weeks of high-fat diet (HFD) feeding. OH: obesity-related hypertension. BW: body weight (**a**). HW: heart weight (**b**). WAT: white adipose tissue. BW, SBP (**f**), and heart rate (**h**) were measured under conscious state. Left ventricular mass (**d**) was measured using the echocardiographic method. The inguinal, perirenal, epididymal, and mesenteric WAT weights were included in the sum of WAT mass (**e**). HW/BW (**c**) means the ratio between heart weight and body weight. Chronic adrenomedullin (ADM, 7.2 μg/kg/day, ip) administration was applied to OH rats for 4 weeks. The Control and OH rats were treated with saline (vehicle). *n* = 8 rats for each group. Values are presented as mean ± SEM. * *p* < 0.05 versus Control (C), and ^#^ *p* < 0.05 versus OH.

HFD feeding for 24 weeks significantly led to obesity and hypertension (Figure 1a,e,f), myocardial hypertrophy (Figure 1b,d), and the reduction of cardiac function in the present study (Table 1). Exogenous ADM application effectively inhibited HFD-induced obesity (Figure 1a,c,d), which was consistent with our recent study [29]. It also significantly reduced heart weight and heart rate and improved hypertension (Figure 1b,e–g). In the present study, HFD feeding significantly caused a decrease in cardiac function and an increase in left ventricular hypertrophy, as demonstrated by echocardiographic analysis that showed obvious decreases in left ventricular ejection fraction (LVEF) and left ventricular fractional shorting (LVFS), but marked increases in left ventricular mass, left ventricular volume at end systole (IVVs), left ventricular internal dimensions at end systole (LVIDs), left ventricular posterior wall dimensions at end diastole (LVPW_d_) and interventricular septum at end diastole (IVS_d_) in HFD-fed rats, but ADM could effectively normalize the cardiac function and left ventricular hypertrophy evidenced by increased LVEF and LVFS, and decreased left ventricular mass, IVVs and LVIDs (Table 1, Figure 1d) after HFD feeding. These results indicate that ADM can effectively improve HFD-induced obesity, hypertension, and myocardial hypertrophy and prevent the deterioration of cardiac function in response to HFD.

### 2.2. Plasma Level of ADM and the Effects of ADM on Metabolic Parameters, Plasma CRP, TNF-α, and T-AOC

**Figure 2:**
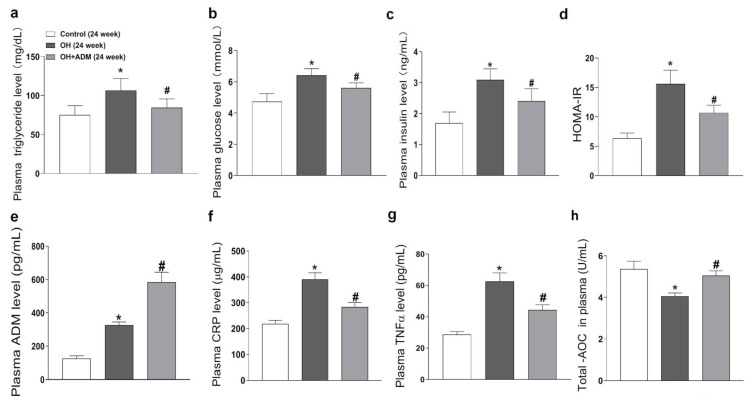
The effects of ADM on plasma metabolic parameters, inflammation markers including C-reaction protein (CRP) and TNF-α, and total antioxidant capacity (T-AOC). The plasma levels of triglyceride (**a**), glucose (**b**), insulin (**c**), ADM (**e**), CRP (**f**), TNF-α (**g**) and T-AOC (**h**) were determined by kits referring to the instructions. At the end of 24 weeks, all rats fasted overnight, and about 1.5 mL of blood was collected from the tail vein. The glucose oxidase method was used to determine the plasma glucose level by using a kit (Jiancheng bioengineering, Nanjing, China). The triglyceride concentration was detected by colorimetric reaction using a kit (Jiancheng bioengineering, Nanjing, China). The enzyme-linked immunoassay (ELISA) method was used to detect the insulin level in plasma by using a kit (RayBiotech, Norcross, GA, USA). The HOMA-IR (homeostasis model assessment of insulin resistance) index was used to assess IR by using the formula: HOMA-IR = [fasting glucose (mmol/L) × fasting insulin (mIU/L)]/22.5 (**d**). *n* = 8 rats for each group. Values are presented as mean ± SEM. * *p* < 0.05 versus Control (C), and ^#^ *p* < 0.05 versus OH.

The levels of triglyceride, glucose, insulin (Figure 2a–c), C-reactionprotein (CRP), TNF-α, and total antioxidant capacity (T-AOC) (Figure 2f–h) in plasma were increased in OH rats, which were markedly decreased by ADM application in HFD-fed rats, as well as HOMA-IR (homeostasis model assessment of insulin resistance, Figure 2d). Moreover, the plasma ADM level was higher in OH rats than in control rats (Figure 2e). ADM intraperitoneal injection further increased the ADM levels in the plasma of OH rats.

### 2.3. Endogenous Expression of ADM and Its Receptor System in Hearts of HFD-Induced OH Rats

**Figure 3:**
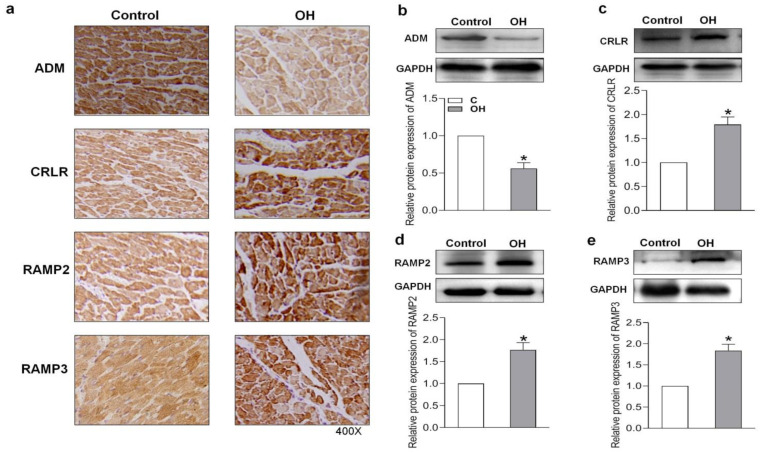
The endogenous ADM, CRLR, RAMP2, and RAMP3 protein expressions after 24 weeks of HFD feeding were determined by immunohistochemical method (**a**) and Western blotting method (**b**–**e**). CRLR, calcitonin receptor-like receptor; RAMP2, receptor activity modifying protein 2; and RAMP3, receptor activity modifying protein 3. *n*  =  4–5 rats for each group. The values are presented as the mean ± SEM. * *p* <  0.05 versus Control (C).

In this study, we examined the endogenous protein expression of ADM and its receptor system in the hearts of HFD-induced OH rats and palmitate (PA)-treated H9c2 cells. Immunohistochemistry and Western blotting results showed that there was a lower protein expression of ADM but higher protein expressions of its receptor system, including CRLR, RAMP2, and RAMP3, in the hearts of OH rats when compared to the control rats (Figure 3a–e).

### 2.4. Effects of ADM on Cardiac Remodeling, Inflammation and Oxidative Stress in HFD-Treated OH Rats

**Figure 4:**
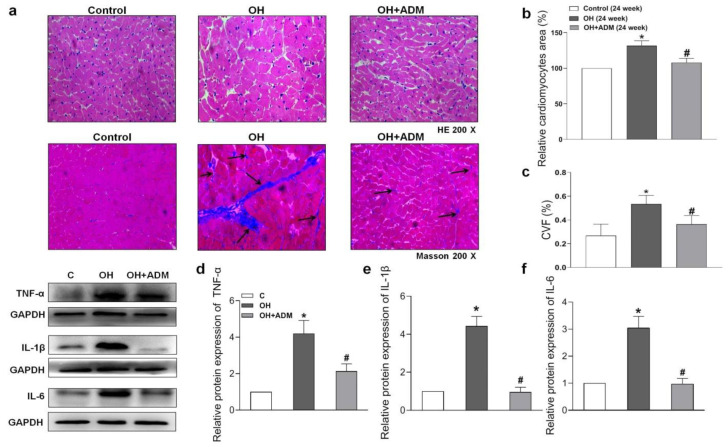
The effects of ADM on remodeling and inflammation in the heart. Representative sections showing the myocardial remolding observed under a microscope (200×). Hematoxylin-eosin (H&E) and Masson’s trichrome-staining (**a**), relative cardiomyocyte area (**b**), and the quantitative myocardial fibrosis analysis (**c**). The arrows indicate the collagen fibers. The typical pro-inflammatory cytokines TNF-α, IL-1β and IL-6 protein expressions (**d**–**f**) in the heart were determined by the Western blotting method. CVF: collagen volume fraction. *n* = 3–8 rats. The values are presented as mean ± SEM. * *p* <  0.05 versus Control (C), and ^#^ *p* <  0.05 versus OH.

**Figure 5:**
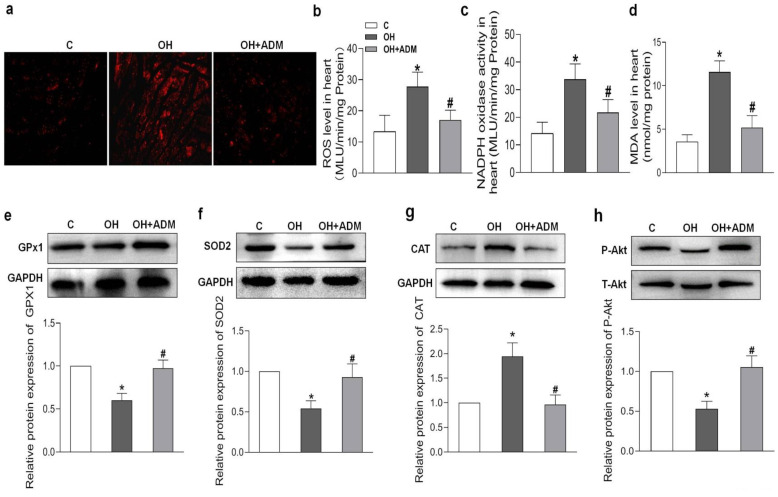
The effects of ADM on cardiac oxidative stress and Akt activation. DHE fluorescence staining for ROS evaluation (**a**), ROS level (**b**) and NADPH activity (**c**) examination by chemiluminescence method, MDA content (**d**) measurement by spectrophotometrical analysis and antioxidant enzymes including GPx1, SOD2, and CAT (**e**–**g**), and Akt phosphorylation (**h**) determination by Western blotting method. C, control; ROS, reactive oxygen species; NADPH, nicotinamide adenine dinucleotide phosphate; MDA, malondialdehyde; GPx1: glutathione peroxidase 1; SOD2, superoxide dismutase 2 (SOD2); and CAT, catalase. *n* = 3–8 rats. The values are presented as the mean ± SEM. * *p* <  0.05 versus Control (C), and ^#^ *p* <  0.05 versus OH.

HFD treatment also markedly induced the increase of cardiomyocyte area and cardiac fibrosis, as demonstrated by H&E staining and Masson staining, which were markedly improved by ADM application in HFD-fed rats (Figure 4a–c). It has been reported that HFD can cause substantial inflammation in the heart, which involves the pathogenesis of cardiac damage and remodeling [1,7]. We first determined the level of protein expression of the typical pro-inflammatory cytokines in the heart. The results showed that TNF-α, IL-1β, and IL-6 protein levels were significantly upregulated in cardiac tissue after HFD, while ADM effectively attenuated the upregulation of these pro-inflammatory cytokines (Figure 4d–f). Studies have indicated that inflammation or hyperlipidemia can enhance ROS production involving cardiac damage and obesity-related complications [30,31]. Nicotinamide adenine dinucleotide phosphate (NADPH) oxidase is a dominant enzyme responsible for ROS production in the heart. HFD can cause an imbalance between the antioxidant system and ROS generation [32]. Malondialdehyde (MDA), a biological marker of oxidative stress, determines the degree of peroxidation of membrane lipids. Glutathione Peroxidase 1 (GPx1), manganese superoxide dismutase, namely superoxide dismutase 2 (SOD2), and catalase (CAT), are antioxidant enzymes involved in ROS scavenging during cardiac injury [33,34]. The decreased GPx1 (Figure 5e) and SOD2 expression (Figure 5f) and increased CAT expression (Figure 5g), ROS level (Figure 5a,b), NADPH oxidase activity (Figure 5c), and MDA content (Figure 5d) caused by HFD were effectively improved by ADM application (Figure 5a–g). These data indicate that ADM involves the improvement of inflammation and oxidative stress in the hearts of OH rats.

### 2.5. Endogenous Expression of ADM and Its Receptor System in PA-Treated H9c2 Cells and the Effects of Exogenous ADM Pretreatment on PA-Induced Inflammation in the H9c2 Cell Line

**Figure 6:**
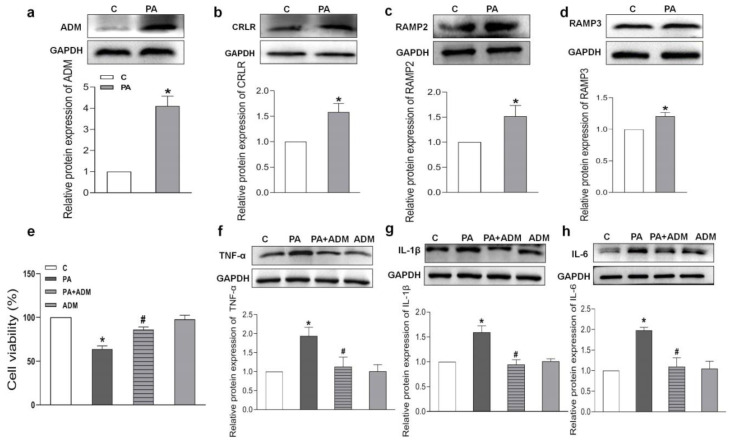
Endogenous protein expressions, cell viability, and the effects of ADM on inflammation caused by palmitic acid (PA) in H9c2 cells. Endogenous protein expressions of ADM, CRLR, RAMP2, and RAMP3 (**a**–**d**) in H9c2 cells treated by PA (200 μM) for 24 h; the roles of PA (200 μM) and ADM (10 nM) alone or their combination in cell viability for 24 h (**e**); and ADM pretreatment in protein expressions of inflammatory cytokines TNF-α, IL-1β and IL-6 induced by PA (**f**–**h**). After ADM (10 nM) pretreatment in the H9c2 cells for 30 min, 200 μM PA was applied for a further 24 h. C, control. *n* = 3–6. The values are presented as the mean ± SEM. * *p* <  0.05 versus Control (C), and ^#^ *p* <  0.05 versus PA.

PA induced higher protein expressions of ADM and its receptor system than those in control H9c2 cells (Figure 6a–d). We wanted to further explore the roles and mechanisms of ADM in inflammation in cardiomyocytes. PA was used to mimic fatty acid stimulation and to verify the roles of ADM in cardiomyocyte damage induced by hyperlipidemia. ADM alone did not cause obvious effects on cell viability, but PA alone significantly decreased it. More importantly, ADM plus PA obviously improved cell viability in the H9c2 cells (Figure 6e). We also observed that ADM in H9c2 cells significantly decreased the protein levels of inflammatory cytokines TNF-α, IL-1β and IL-6 induced by PA (Figure 6f,g). These results confirmed the protective roles of ADM in fatty acid-induced cardiomyocyte viability and inflammation.

### 2.6. Effects of ADM Pretreatment on Oxidative Stress in PA-Treated H9c2 Cells

**Figure 7:**
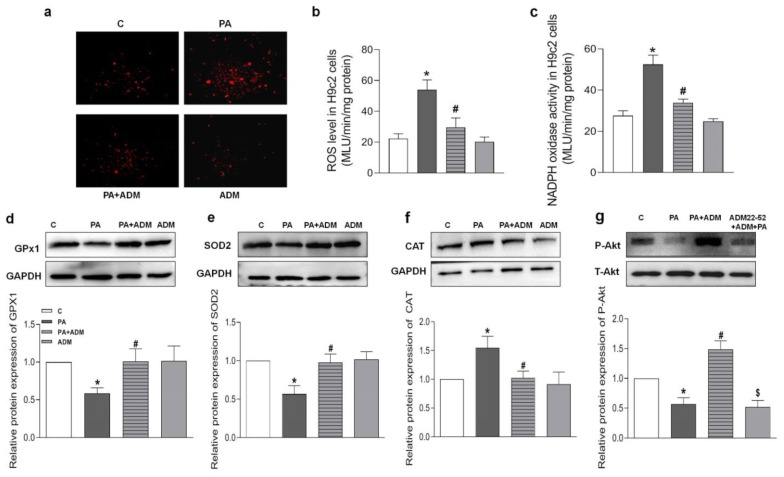
The effects of ADM on PA-induced oxidative stress and decreased Akt actiation in H9c2 cells. DHE fluorescence staining for intracellular ROS evaluation (**a**) in H9c2 cells, ROS level (**b**) and NADPH activity (**c**) were examined in H9c2 cells by chemiluminescence method, antioxidant enzymes including GPx1, SOD2, and CAT (**d**–**f**), and Akt phosphorylation (**g**) were analyzed in H9c2 cells by Western blotting method. The H9c2 cells were pretreated with 10 nM ADM for 30 min, subsequently treated by PA (200 μM) for 24 h. *n* = 3–6. The values are presented as the mean ± SEM. * *p* <  0.05 versus Control (C), ^#^ *p* <  0.05 versus PA, and ^$^ *p* <  0.05 versus PA+ADM.

Since ADM in vitro attenuated the inflammatory response [20,21], it is speculated that ADM application may exert a beneficial effect on PA-caused oxidative stress in cardiomyocytes. The results in Figure 7a–c show that the increased ROS level and NADPH oxidase activity caused by PA were notably reduced after ADM treatment in H9 c2 cells, demonstrating the antioxidant effects of ADM in vitro. Moreover, PA-treated H2 cells showed reduced GPx1 and SOD2 protein expressions, and increased CAT expression, but the exogenous ADM pretreatment restored their protein expressions to normal levels in PA-stimulated H9c2 cells (Figure 7d–f), which further confirmed that ADM effectively improved oxidative stress.

### 2.7. Roles of ADM22-52 (ADM Receptor Antagonist) and A6730 (Akt Activation Inhibitor) in ADM’s Responses to PA-Caused Inflammation and Oxidative Stress in H9c2 Cells

**Figure 8:**
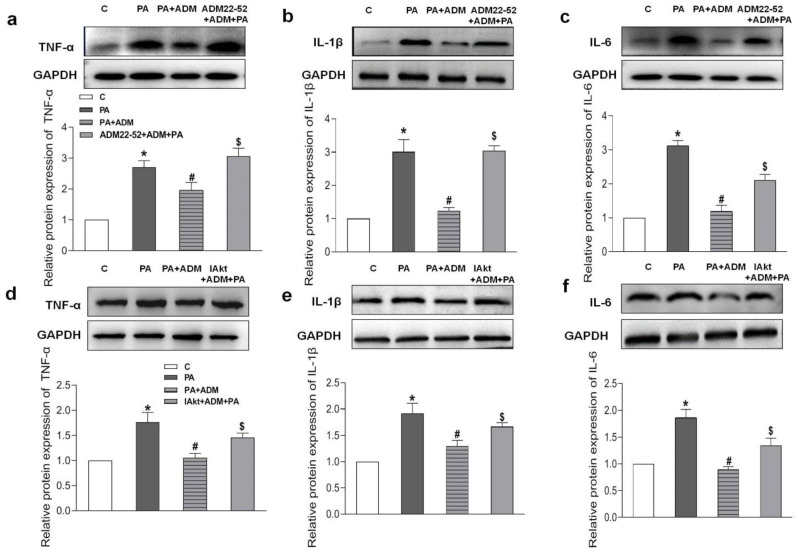
The effects of ADM receptor antagonist ADM22-52 (10^−6^ M, **a**–**c**) or Akt activation inhibitor A6730 (IAkt, 10 μM, **d**–**f**) on ADM’s response to pro-inflammatory cytokines TNF-α, IL-1β and IL-6 protein expressions caused by PA in H9c2 cells. ADM (10 nM) was administrated in H9c2 cells for 30 min; subsequently, cells were exposed to PA (200 μM) for further 24 h. ADM22-52 or A6730 was added 30 min before ADM application. *n* = 3–5. The values are presented as the mean ± SEM. * *p* <  0.05 versus Control (C). ^#^ *p* <  0.05 versus PA, and ^$^ *p* <  0.05 versus PA+ADM.

**Figure 9:**
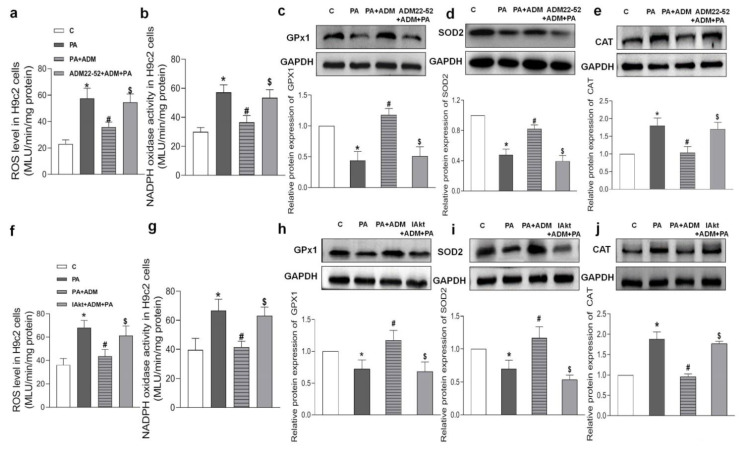
The roles of 10^−6^ M ADM22-52 (ADM receptor antagonist. (**a**–**e**) or 10 μM A6730 (Akt activation inhibitor, IAkt. (**f**–**j**) in ADM’s response to PA-induced oxidative stress, including ROS level (**a**,**f**) and NADPH activity (**b**,**g**), and antioxidant enzymes GPx1, SOD2, and CAT protein expressions (**c**–**e**,**h**–**j**) in H9c2 cells. ADM (10 nM) was applied in H9c2 cells for 30 min, then cells were treated by 200 μM PA for a further 24 h. ADM22-52 or A6730 was added 30 min before ADM application. *n* = 3–6. The values are presented as the mean ± SEM. * *p* <  0.05 versus Control (C). ^#^ *p* <  0.05 versus PA, and ^$^ *p* <  0.05 versus PA+ADM.

Increasing evidence indicates that the receptor–Akt pathway involves the protective effects of ADM on some diseases [20,35,36]. Therefore, we examined whether the ADM receptor antagonist ADM22-52 and Akt activation inhibitor A6730 could block the ADM’s effects on PA-induced inflammation and oxidative stress in H9c2 cells, and whether ADM could promote Akt activation. The data in Figure 5h showed that there was a decreased phosphorylated Akt (P-Akt) level in the heart tissue of the OH group when compared to the Control group, but the P-Akt level was higher in the OH+ADM group than in the OH group. Similarly, we observed decreased P-Akt levels in PA-stimulated H9c2 cells, whereas ADM treatment could also restore P-Akt levels in PA-stimulated H9c2 cells (Figure 7g). Then, we found that the ADM receptor antagonist ADM22-52 or Akt inhibitor A6730 effectively reversed exogenous ADM-caused anti-inflammatory (Figure 8a–f) and antioxidant effects (Figure 9a–j) in PA-stimulated H9c2 cells. Furthermore, ADM22-52 also prevented Akt activation caused by ADM (Figure 7g). Therefore, these results demonstrate that the protective effects of ADM on cardiac inflammation and oxidative stress may involve the activation of the receptor-Akt pathway, and the receptor-Akt pathway contributes to the inhibitory effects of ADM on inflammation and oxidative stress in cardiomyocytes.

## 3. Discussion

The primary novel findings in this study were that ADM contributed to the improvement of heart structure and function, lowered BP, and reduced inflammation and oxidative stress in the hearts of rats with OH induced by HFD. ADM could also attenuate PA-caused inflammation and oxidative stress responses in cardiomyocytes, which were associated with the receptor-Akt pathway.

It has been reported that there is an increased plasma ADM level in people with obesity [24,25], and ADM might derive from many other organs or tissues, such as WAT. This increase could be a compensatory response of the body. In this study, there was an obvious elevation in plasma ADM level, but its protein level was decreased in the heart, and its receptor system including CRLR, RAMP2, and RAMP3 levels were increased. The results suggest that the ADM and ADM receptor systems may be involved in cardiac remodeling and function in OH rats. The increased ADM receptor expression and decreased ADM expression in the heart may be important in the cardiac abnormality in OH. ADM may exert protective effects on the heart in the OH state, and its deficiency may be associated with deterioration in cardiomyopathy in response to HFD. In this study, our findings confirmed that ADM exerted its beneficial roles in heart structure and function in OH rats, and ADM receptors mediated its effects in cardiomyocytes.

To adapt to volume overload and/or mechanical stress of pressure during OH, the heart gradually becomes compensatory hypertrophy, which is featured by an increment in LV mass without ventricular function improvement, but this hypertrophy can eventually decompensate [1,3,8]. In this study, we found that the heart in the OH group had obvious hypertrophy, as shown by the increased HW, left ventricular mass, IVVs, and LVIDs, and decreased heart function, as shown by the decreased LVEF and LVFS. Mechanisms underlying heart abnormality in OH patients are related to the altered fat tissue amount, hypertension, inflammatory, and oxidative stress processes, and changed cardiac physiology [3,6,7]. There are many findings involving hypotensive, anti-inflammatory, and antioxidant effects of ADM [9,21,22,23,37]. For instance, ADM has been shown to inhibit oxidative stress and inflammation in vascular endothelial cells [23]. We previously reported the protective effects of ADM in the WAT of obese rats [20,29]. For instance, ADM could reduce inflammation in WAT in obese rats induced by HFD [29], and we recently also found that ADM could improve IR in WAT by inhibiting inflammation and oxidative stress [20]. In this study, we evidenced that administration of ADM not only lowered BP, but also reduced TG, TNF-α and CRP levels in plasma. Moreover, ADM improved the total antioxidant capacity in the plasma of OH rats. These results showed that ADM improved cardiac remodeling and function, which may be due to its hypotensive, anti-inflammatory, and antioxidant effects. These beneficial effects may effectively prevent deterioration in cardiac structure and function in the OH state.

The local effects of ADM on inflammation and oxidative stress in the heart were also explored. It is also known that the increased amount of visceral adipose tissue can cause elevated levels of plasma fatty acids, which lead to more intensive pro-inflammatory cytokines and ROS production [4,6,7,38,39,40,41,42,43] and reduce antioxidant enzyme activity such as GPx1, SOD2, and CAT [39,43]. Pro-inflammatory cytokines and ROS in neighboring cardiomyocytes can synergically contribute to pathological hypertrophy, which activates the inflammatory signaling pathways and suppresses the Akt-mTOR pathway [11,39,40,43,44]. In the present study, HFD feeding appeared to increase inflammatory cytokines (TNF-α, IL-1β and IL-6) generation and oxidative stress demonstrated by a high content of MDA, which is a by-product of lipid peroxidation and is regarded as a crucial marker of oxidative stress. ROS level, NADPH oxidase activity, and altered antioxidant enzymes (GPx1, SOD2, and CAT) expression in cardiac tissues. ADM application effectively inhibited local inflammation and oxidative stress and restored antioxidant enzyme expression levels in the heart. Therefore, ADM may be an effective active peptide for attenuating the adaptive structure response and cardiac injury caused by local excessive inflammation and oxidative stress in the heart.

It is known that the effects of ADM are mostly mediated by ADM receptors [13,15]. Previous studies indicated that ADM exerts protective roles in numerous cell types via Akt activation, such as cardiomyocytes, vascular endothelial cells, endothelial progenitor cells, rat leydig cells, and differentiated adipocytes [20,45,46,47,48]. Referring to these findings, it can be hypothesized that ADM has protective roles in the heart of the OH condition via the receptor-Akt pathway. According to our in vivo and vitro data, we found that the phosphorylated Akt (P-Akt) level was downregulated in the heart of OH rats or PA-treated H9c2 cells, whereas ADM application led to an enhanced phosphorylation level of Akt under HFD treatment in rats or PA treatment in H9c2 cells. The receptor antagonist ADM22-52 effectively blocked the ADM’s action on the P-Akt in PA-treated H9c2 cells. Moreover, ADM22-52 and Akt activation inhibitor A6730 pretreatment also attenuated the anti-inflammatory and antioxidative effects of ADM in PA-treated H9c2 cells. These results suggest that the protective roles of ADM in the heart involve receptor-Akt signaling pathway activation.

A decrease in BW, especially in WAT, may substantially change heart muscle remodeling, function, and symptoms connected to obesity. In this study, it was obvious in cardiac hypertrophy and fibrosis, and there was an obvious decrease in cardiac function, whereas the cardiac remodeling and function were almost normalized after ADM application. The reduction in body mass and in WAT after ADM application may also involve the normalization of BP and the improvement of left ventricular remodeling and function. ADM-induced inhibition of food intake [29] and the promotion of lipolysis [49] may be responsible for BW and WAT loss. Therefore, the improvement of cardiac remodeling and the normalization of cardiac function may be associated with the reduction of cardiac workload and the inhibition of inflammatory and oxidative stress processes. ADM also has a positive impact on the regulation of metabolic processes, such as hyperlipemia and IR in WAT, as demonstrated by our previous study [20,29]; therefore, ADM may have positive effects on cardiac tissue indirectly through the improvement of hyperlipemia and IR.

## 4. Methods

### 4.1. Animals

Male SD rats weighing 300 g or so at the age of 2 months were chosen for the study. They were randomized into the Control and HFD groups. Rats (*n* = 36) were housed in a room that possessed a 12 h light-dark cycle and controllable temperature and humidity. They can be permitted access to tap water and rat chow ad libitum. The obese group (*n* = 28) was fed an HFD (fat provided 45% kilocalories; Nantong, China) for 20 weeks, and the control group (*n* = 8) was fed a normal low-fat diet (fat provided 12% kilocalories; Nantong, China). The experiments complied with the Guidelines for the Care and Use of Laboratory Animals (NIH publication, 8th edition, 2011) and were approved by the animal research ethics committee of Nanjing Medical University (1911016, 14 September 2020). The criterion for the OH rats was that the BW was 20% higher than that of the mean weight of the control rats, and the SBP was more than or equal to 140 mmHg after 20 weeks of HFD feeding. OH rats (*n* = 16) were further randomized into two groups and continued to receive an HFD for 4 weeks. An intraperitoneal injection of ADM (7.2 μg/kg/day) was applied to the OH rats (*n* = 8), and the remaining rats (*n* = 8) received an equal volume of saline. At the end of 24 weeks, the BW was measured, and the visceral WAT was also weighted after rats were anesthetized with sodium pentobarbital.

### 4.2. Measurement of Heart and SBP

The heart rate and SBP of the rat tail artery in waking state were measured by a computerized tail-cuff system (NIBP, ADInstruments, Sydney, New South Wales, Australia). To obtain a steady pulsation of the tail artery, the rat was warmed for 15 min or so and was trained for SBP detection for 10 days before the formal experiment began. The values were obtained by averaging 10 measurements per rat [50].

### 4.3. Echocardiographic Measurement

Ketamine was used to anesthetize the rats, and then a Vevo 2100 (VisualSonics, Toronto, Ontario, Canada) system was used to evaluate cardiac function. Left ventricular mass, IVVs, LVIDs, LVPW_d,_ IVS_d,_ LVEF and LVFS were measured [51].

### 4.4. Cardiac Sample Preparation and H&E and Masson Staining

Rats were anesthetized with 2% sodium pentobarbital solution according to the weight (0.3 mL/100 g) and the heart was removed quickly. One part of the anterior wall of the left ventricle tissue was kept at −80 °C for further analysis, and the other was incubated overnight in formalin. Cardiac tissue slices were cut. The sections of cardiac tissue slices (6~8 μm) received hematoxylin-eosin (H&E) and Masson’s trichrome staining. The images were obtained with a light microscope. The cardiomyocyte area was used to evaluate the degree of pathological changes in myocardial hypertrophy. Cardiac fibrosis was evaluated by Masson’s trichrome staining. Five fields of each slice were selected randomly, and Image-Pro Plus 6.0 was used to assess the collagen volume fraction (CVF) [51].

### 4.5. Enzyme-Linked Immunosorbent Assay (ELISA)

The plasma levels of ADM, CRP, and TNF-α were determined by the ELISA method using the kits referring to the manufacturer’s instructions [20]. The respective final solution was detected using a microplate reader (ELX800, BioTek, Winooski, VT, USA) at a certain wavelength. The ELISA kit for ADM was purchased from Phoenix Pharmaceuticals (Burlingame, CA, USA), and the kits for CRP and TNF-α were obtained from RayBiotech (Peachtree Corners, GA, USA).

### 4.6. Cell Culture and Treatment

The embryonic rat heart-derived H9c2 cell line was from Costar Corning Inc. (Corning, CA, USA). DMEM/F12 medium supplemented with 10% fetal bovine serum and 1% penicillin (100 U)/streptomycin (100 mg/mL) was used to culture the H9c2 cells. H9c2 cells were treated with 200 μM PA binding BSA at a ratio of 5:1 for 24 h to mimic high lipid stimulation for inducing inflammation and oxidative stress. PA was applied 30 min after 10 nM of ADM pretreatment. ADM receptor antagonist ADM22-52 (1 μM) or Akt inhibitor A6730 (10 μM) was added 30 min before ADM pretreatment. The phosphorylated protein level of Akt was detected 30 min after PA application.

### 4.7. Cell Viability Assay

The H9c2 cell suspension (100 μL) was placed into a 96-well plate and cultured in a cell incubator for 24 h at 37 °C. The effect of ADM (10 nM) alone, PA (200 μM) alone or ADM plus PA on cell viability was determined by Cell Counting Kit-8 (CCK8) cell cytotoxicity test. The test substances (10 μL) were added to the plate. After incubation, the CCK8 solution (10 μL) was applied to each well and incubated for 3 h. A microplate reader (ELX800; BioTek, Winooski, VT, USA) was used to determine the absorbance at 450 nm.

### 4.8. DHE Fluorescence Staining for ROS Level Assay

ROS production in myocardium or H9c2 cells was evaluated with DHE staining. A part of the left ventricle was excised from the whole heart, rinsed in ice-cold PBS, and then incubated overnight in formalin. The heart tissue was embedded in an OCT compound before DHE staining. The tissue was cut into 25 μm thick sections that were incubated with 10 μM DHE in PBS for 5 min in a dark and humidified container at 37 °C. Under the same conditions, the H9c2 cells (3 × 10^5^ cells/mL) were incubated by PBS containing 10 μM of DHE in six-well plates. Then, they were rinsed three times with cold PBS. Under excitation at 518 nm and emission at 605 nm, fluorescence was detected by fluorescence microscopy (DP70, Olympus Optical, Tokyo, Japan).

### 4.9. Analysis of Inflammation and Oxidative Stress Markers

We analyzed the state of inflammation and the antioxidant system in the plasma, heart tissue, and H9c2 cells. The T-AOC was determined using an analysis kit (Jiancheng Bioengineering Institute, Nanjing, China). The state of inflammation was determined by the protein expression levels of pro-inflammatory cytokines TNF-α, IL-1β and IL-6 using the Western blot method. We also evaluated the protein expression of the antioxidant enzymes system, including GPx1, SOD2, and CAT, with the Western blot method. Lipid peroxidation was assessed by measuring MDA concentration using a kit (Jiancheng Bioengineering Institute, Nanjing, China). Lipid peroxidation was measured by the reaction of MDA with thiobarbituric acid to form a colorimetric product that is proportional to the MDA content. The color intensity was detected spectrophotometrically at 532 nm.

### 4.10. Determination of ROS Level and NADPH Oxidase Activity

NADPH oxidase is one of the dominant mechanisms of ROS production in the heart. Therefore, we additionally determined the ROS amount and NADPH oxidase activity in heart muscle and H9c2 cells by using the enhanced lucigenin-derived chemiluminescence method [26]. Both dark-adapted 5 μM lucigenin and 100 μM NADPH was used to trigger the photon emission, and the background chemiluminescence was measured in the buffer containing lucigenin (5 μM). The data from a luminometer (Turner, CA, USA) were averaged by using 10 measurements in 10 min and were presented as the mean of light unit (MLU)/min/mg protein. The level of ROS in myocardium or H9c2 cells was detected by the lucigenin-derived chemiluminescence method, referring to previously published literature [32,50].

### 4.11. Immunohistochemistry

Immunohistochemistry was utilized to detect ADM and its receptor system expression in the hearts of the Control and OH rats. Primary antibodies (1:100), including ADM, CRLR, RAMP2, and RAMP3, and horseradish peroxidase-conjugated goat anti-rabbit antibody were used. A analytical reagent, 3,3-diaminobenzidine, was used to show the positive cells. Images were taken from a light microscope (BX-51, Olympus, Tokyo, Japan).

### 4.12. Western Blot Analysis

Cardiac tissues or H9c2 cells were sonicated and homogenized in RIPA lysis buffer. After the protein was quantified, polyacrylamide gel electrophoresis (PAGE) (Bio-Rad) was used to separate the equal quantities of tissues or H9c2 cell protein lysates, which were further electrotransferred onto polyvinylidene difluoride membranes. Primary antibodies against ADM, CRLR, RAMP2/3, TNF-α, IL-1β, IL-6, SOD2, GPx1, CAT, total-Akt (T-Akt), phosphorylated-Akt (P-Akt), or GAPDH were used overnight in 4 °C fridge, followed by incubation with appropriate secondary antibodies. The intensity of the protein bands was normalized with total Akt or GAPDH levels. The Odyssey Imaging System (LI-COR Biosciences, Lincoln, NE, USA) was used to quantify the signals, which were further quantified by Image J software for determining the amount of detected protein.

### 4.13. Reagents and Antibodies

Rat The ADM (molecular formula: C_242_H_381_N_77_O_75_S_5_) was obtained from Bachem (Bubendorff, Switzerland). PA was purchased from Sigma-Aldrich. ADM receptor antagonist ADM22-52 and Akt inhibitor A6730 were purchased from Anaspec (Fremont, CA, USA). DMEM, 0.25% trypsin-EDTA, fetal bovine serum, trypsin, and streptomycin/penicillin were from Thermo Fisher Scientific (Pudong New District, Shanghai, China). The antibodies of ADM, CRLR, RAMP2, RAMP3, and TNF-α were obtained from Affinity Biosciences (Pottstown, PA, USA). The antibodies of IL-6, IL-1β, SOD2, GPx1, CAT, and GAPDH were from Proteintech (SANYING, Wuhan, China). Total Akt and phosphorylated Akt were purchased from Cell Signaling Technology (Shanghai, China).

### 4.14. Statistics

In this study, GraphPad Prism version 8.0.2 (San Diego, CA, USA) software was used to analyze all data. The differences in the mean values were evaluated by unpaired *t* test between the two groups. For more than two groups, one-way or two-way ANOVA was used for data analysis, which was further analyzed by Bonferroni post hoc analysis. The data were presented as mean ± SEM (standard error of the mean). A *p* < 0.05 was considered statistically significant.

## 5. Conclusions

In this study, based on the animal model, we report that HFD per se may be the main driver by increasing WAT mass and BP, and regulating inflammation and oxidative stress in the heart muscle. Exogenous ADM application efficiently normalized the heart remolding and function in OH rats. The mechanisms of ADM’s action may be through improving hypertension and inhibiting cardiac inflammation and oxidative stress via the activation of the receptor-Akt pathway. The improvement of hyperlipemia and IR and the reduction in BW and in WAT may also be involved.

## Figures and Tables

**Table 1:** Left ventricle dimension and function measurement in rats by echocardiography after 24 weeks of diet.

Parameters	Control	OH	OH+ADM
LVVs (μL)	107 ± 10.8	161 ± 12.3 *	124 ± 12.5 ^#^
LVIDs (mm)	4.76 ± 0.54	5.80 ± 0.65 *	5.09 ± 0.58 ^#^
LVPW_d_ (mm)	1.92 ± 0.06	2.29 ± 0.09 *	2.15 ± 0.16
IVS_d_ (mm)	1.94 ± 0.08	2.26 ± 0.13 *	2.12 ± 0.07
LVEF (%)	74.7 ± 3.59	59.1 ± 2.48 *	69.1 ± 1.67 ^#^
LVFS (%)	44.1 ± 2.56	35.3 ± 2.09 *	43.4 ± 1.35 ^#^

A control diet (12% kcal as fat, Control) or a high-fat diet (HFD, 45% kcal as fat) for inducing obesity-related hypertension (OH) was used to feed male Sprague Dawley (SD) rats for 20 weeks. Saline (vehicle) or ADM (7.2 μg/kg/day, *n* = 8) by intraperitoneal injection were applied to the Control and OH rats, and HFD was further fed for 4 weeks. Abbreviations: LVVs, left ventricular volume at end systole; LVIDs, left ventricular internal dimensions at end systole; LVPW, left ventricular posterior wall dimensions at end diastole; IVS, interventricular septum at end diastole; LVEF, left ventricular ejection fraction; LVFS, left ventricular fractional shorting; ADM, adrenomedullin. *n* = 8 for each group. Values are presented as mean ± SEM. * *p* < 0.05 versus Control, and ^#^ *p* < 0.05 versus OH.

## Data Availability

Data is contained within the article.

## Abbreviations

OH	obesity-related hypertension
TNF-α	tumor necrosis factor-α
IL-1β	interleukin-1β
IL-6	interleukin-6
ROS	reactive oxygen species
Akt	protein kinase B
ADM	adrenomedullin
CRLR	calcitonin receptor-like receptor
RAMP2	receptor activity modifying protein 2
RAMP3	receptor activity modifying protein 3
HFD	high-fat diet
BP	blood pressure
LVEF	Left ventricular ejection fraction
LVFS	fractional shortening
IVVs	left ventricular mass, left ventricular volume at end systole
LVIDs	left ventricular internal dimensions at end systole
LVPWd	left ventricular posterior wall dimensions at end diastole
IVSd	interventricular septal thickness at the end of diastole
SBP	systolic blood pressue
BW	body weight
HW	heart weight
WAT	white adipose tissue
HW/BW	the ratio between the heart weight and body weight
CRP	C-reactionprotein
T-AOC	total antioxidant capacity
HOMA-IR	homeostasis model assessment of insulin resistance
NADPH	Nicotinamide adenine dinucleotide phosphate
MDA	malondialdehyde.
GPx1	glutathione peroxidase
SOD2	superoxide dismutase 2
CAT	catalase
CVF	collagen volume fraction
PA	palmitate
